# Competition Between Object Topology and Surface Features in Children’s Extension of Novel Nouns

**DOI:** 10.1162/opmi_a_00073

**Published:** 2023-04-05

**Authors:** Praveen Kenderla, Sung-Ho Kim, Melissa M. Kibbe

**Affiliations:** Department of Psychological & Brain Sciences, Boston University, Boston, MA; Department of Psychology, Ewha Womans University, Seoul, South Korea

**Keywords:** topology, inference, word learning, object cognition, categorization

## Abstract

Objects’ topological properties play a central role in object perception, superseding objects’ surface features in object representation and tracking from early in development. We asked about the role of objects’ topological properties in children’s generalization of novel labels to objects. We adapted the classic name generalization task of Landau et al. (1988, 1992). In three experiments, we showed children (*n* = 151; 3–8-year-olds) a novel object (the standard) and gave the object a novel label. We then showed children three potential target objects and asked children which of the objects shared the same label as the standard. In Experiment 1, the standard object either did or did not contain a hole, and we asked whether children would extend the standard’s label to a target object that shared either metric shape or topology with the standard. Experiment 2 served as a control condition for Experiment 1. In Experiment 3, we pitted topology against another surface feature, color. We found that objects’ topology competed with objects’ surface features (both shape and color) in children’s extension of labels to novel objects. We discuss possible implications for our understanding of the inductive potential of objects’ topologies for making inferences about objects’ categories across early development.

## INTRODUCTION

The ability to rapidly categorize objects allows us to efficiently store and retrieve large amounts of information and to make inferences about objects that we have never seen before (Gelman & Meyer, 2011). For example, a toddler learning that “ball” refers to “a round toy that can be thrown, rolled, and caught” can immediately refer to other ball-like objects that they never encountered before as “ball” and can infer those objects’ categories and functions. Object categorization is thus a powerful means for children to efficiently learn about and interact with the world around them.

To categorize objects, children must treat objects of a category as similar in some way, and an object’s shape can be an important cue in forming categories and learning new words. Evidence for this comes from studies that show that children extend labels of objects to other objects that are similar in shape over other surface features like color, texture, or size, a phenomenon known as “shape bias” (Diesendruck & Bloom, 2003; Landau et al., 1988, 1992; Smith et al., 1996). Shape bias is often examined using the name generalization task (Booth & Waxman, 2008; Diesendruck & Bloom, 2003; Landau et al., 1988, 1992, 1998; see Colunga & Smith, 2008; Elman, 2008; Kucker et al., 2019; Markson et al., 2008, for reviews). A single trial of the name generalization task proceeds as follows. An experimenter presents an object (the standard) and gives the object a novel label (e.g., “Look, this is a *toma*!”). The experimenter then presents three test objects that each share one feature with the standard object (but differ from the standard in their other features): for example, one is the same shape, one is the same texture, and one is the same color as the standard object. The experimenter asks children to point to the object that they think would have the same label as the standard (e.g., “Which one of these is also a *toma*?”). Children are more likely to select the object that shares the same shape as the standard over objects that share other surface features (like color or texture), suggesting that an object’s shape is rapidly associated with a count noun, and the extension of the noun is taken to refer to objects with similar shape (Diesendruck & Bloom, 2003; Landau et al., 1988, 1992).

There have been a number of controversies about the interpretations of the shape bias: Whether this bias results from the perceptual or conceptual salience of shape; whether it is specific to this name extension task; whether it is a consequence of learning object names; and how it is associated with knowledge of object function (e.g., Booth & Waxman, 2002; Booth et al., 2005; Diesendruck & Bloom, 2003; Landau et al., 1998; Smith, 1995, 1999; Soja et al., 1991; Zuniga-Montanez et al., 2021; see the General Discussion section below for our discussion of these in the context of the current research). Despite these controversies, the idea that shape plays a critical role in object categorization is widely accepted. Object kinds, particularly those at the basic level, tend to have similar shapes (e.g., Rosch, 1978; Rosch et al., 1976), and thus sensitivity to shape may help children make ecologically valid generalizations of new labels. Related to this, shape can further provide strong inductive potential to the categories of artifacts, which children may use as a proxy for the object’s function (Nelson et al., 2000; Ware & Booth, 2010; but see also Landau et al., 1998). Most importantly, the shape of rigid objects is relatively stable across different contexts while other surface features may vary depending on lighting conditions. If a caregiver holds up and moves a rigid object in different ways during naming, an infant will be able to able to abstract invariant shape across different views of the same object undergoing different transformations, and this invariant shape could be useful for children learning to name objects, as they could more easily extend the labels they learn to similar objects they encounter (Gogate & Hollich, 2010).

Despite its importance in object categorization, however, shape is a complex concept and what aspect of shape is invariant can depend on the level of description in both perception and geometric theories (i.e., what is invariant in one level of description can be not invariant in a different level). In this study, we questioned what geometric properties (invariances) can influence children’s judgments of which objects have the “same shape”, which can therefore be referred to with the same label. To answer to this question, we were interested in two different geometrical approaches to shape, Euclidean geometry and topology, in the context of the name generalization task. According to the mathematician Klein’s transformational geometry framework, theories of geometry and their associated shape properties can be classified into a hierarchy with increasing levels of abstraction (i.e., Klein hierarchy), in terms of their geometrical transformations (i.e., invariant transformations) under which certain geometric properties remain unaltered (Klein, 1893). Thus, the notion of shape can be defined by the properties that are stable under different geometrical transformations (see Todd & Petrov, 2022 for a review).

In classical Euclidean geometry, there are four types of invariant transformations, called similarity transformations: translation, rotation, reflection, and uniform dilation (resizing).^1^ These transformations alter an object’s position, orientation, handedness, and size, respectively, but preserve its angle and length ratios (which are properties associated with people’s intuitive notion of shape); thus, differences in objects’ location, orientation, handedness, and size are irrelevant, but variability in properties of angle and length ratio are relevant in characterizing objects for Euclidean geometry. In other words, a large square and a small square in different orientations and locations are considered equivalent, i.e., having the same Euclidean (or metric) shape.

If people represent forms according to their Euclidean properties, or people’s intuitive sense of geometry corresponds to Euclidean geometry, they would be sensitive to transformations which alter metric shape, but not sensitive to transformations of the properties that are irrelevant to defining objects in Euclidean geometry, such as position, orientation, and size. The notion of Euclidean shape indeed seems consistent with the findings of the shape bias in literature such as Landau et al.’s original study and many others, which showed that children extend object labels despite size changes of the same Euclidean shape, but not to changes in angular properties of the objects (e.g., from a “U”- to a “L”-shaped object) (e.g., Landau et al., 1988; Landau & Leyton, 1999).

In spite of metric shape’s stability under various geometric transformations, there are more abstract shape properties like structural properties describing relations between object parts (Hoffman & Richards, 1984; Hummel & Biederman, 1992; Marr & Nishihara, 1978), which survive changes in viewpoints and can provide stability to perceptual representations in the face of changes in the environment. When making perceptual judgments about forms, these abstract cues tend to take priority over variations in metric shape (Amir et al., 2014; Lazareva et al., 2008; Lowet et al., 2018). In the Klein hierarchy of geometry, the most abstract level is topology. Topology can be conceptualized by its invariant transformations, often called “rubber-sheet” transformations, which include continuous deformation of objects such as twisting, stretching, and bending; however, they do not include tearing an object in two, poking holes in an object, or gluing two objects together. Topological properties—the number of holes, connectedness, and inside/outside relationship of an object—remain invariant under rubber-sheet deformations, and these invariants remain unaltered under the transformations of all lower-level geometries (i.e., projective, affine, and Euclidean geometries) as well, but not vice versa, making it the most abstract form of geometry (Todd & Petrov, 2022, for review). Owing to the generality of topological transformations, two objects are topologically equivalent if they contain the same number of holes, even if they have distinct shapes. For example, a cup with a handle and a donut are topologically equivalent because they each contain a single hole, and a solid sphere and a solid triangle are topologically equivalent because they each contain no holes; but a donut is not equivalent to a sphere because it is not possible to continuously deform a sphere into a donut.

Visual perception research has revealed that objects’ topological properties, as stable structural properties, can supersede objects’ surface features (including metric shape) in various visual tasks involving object representations in adults, such as the perception of apparent motion (Chen, 1985; Zhuo et al., 2003), numerosity perception (He et al., 2015), visual working memory (Wei et al., 2019), and multiple object tracking (Zhou et al., 2010). For example, adults can discriminate topologically distinct objects significantly faster than objects that differ in metric shape (Chen, 1985). And in a multiple object tracking task, adults’ accuracy to track items decreased significantly when they tracked objects that changed in topological properties as they moved (e.g., a target object with one hole changed to have two holes or no holes), but their performance was not hampered when they tracked objects that morphed in shape as they moved (e.g., a target object changed from triangle to square; Zhou et al., 2010). Compared to changes in metric shape, topological changes to objects are highly salient and more readily detected (Todd et al., 2014). Each of these studies provides evidence that the visual system is able to abstract invariant topological features from objects that have very different metric properties, such as size and shape, and this information is then used by the visual system in various object-related processing, such as forming persistent object representations and computing motion correspondence.

Objects’ topological properties also are fundamental to object perception and cognition early in development (Chien et al., 2012; Turati et al., 2003). Chien et al. (2012) showed that infants by around 1 month of age discriminate objects based on topological properties, but not until 3.5 months do infants discriminate different shapes, suggesting that infants process topological properties earlier in development than surface features. Infants’ expectations about how objects should interact is constrained by topological properties (for example, infants have different expectations about what can happen to an object that hides inside a tube compared to behind an occluder; Baillargeon et al., 2012; Hespos & Baillargeon, 2001a, 2001b, 2006; Wang et al., 2005), and, like adults, infants’ object tracking is impacted by changes in objects’ topologies (Kibbe & Leslie, 2016). By at least 3 years, children also can explicitly identify and count holes in objects (Giralt & Bloom, 2000), suggesting a role for objects’ topology in higher-level processing early in childhood.

Given the primacy of topology over surface features like metric shape for object representation, and given that previous work showed that children have a strong bias toward object shape when categorizing objects, here we asked what role topology might play in children’s inferences about the extension of novel nouns. Because topological properties are fundamental to the *invariant structure* of an object, these properties could carry different information about objects than shape or other surface features. Like shape, topological properties can be diagnostic of an artifact’s category or function (e.g., despite their similar shapes, a needle can be threaded while a pin cannot). Topological properties may also be associated with labels in the real world. For example, despite changes in shape, the label ‘ball’ can be used to an inflated ball and a deflated ball. Topological properties of objects also impact the way humans interact with objects even when those objects have similar functions. For example, to take a drink we may grip the (topologically open) handle of a mug, but the (topologically closed) outside of a cup. Thus, topological properties of objects may play a different role in inferences about objects’ categories than shape does. And because topological properties play a more primary role in object representation than surface featural properties such as shape, they may compete with or even supersede surface features as the relevant object properties in children’s inferences about the relevant perceptual dimensions to which a novel label refers.

In this study, we examined the role of topological properties in 3–7-year-old children’s extension of novel labels to objects. In three experiments, we used the classic name generalization task that has consistently revealed children’s shape bias (Diesendruck & Bloom, 2003; Landau et al., 1988, 1992). In Experiment 1, we manipulated the topology of the standard object (by manipulating whether or not the object had a hole), gave it a novel label, and then asked children to choose from three test objects which object shared the same label: an object with the same metric shape but different topology than the standard, an object with the same topology but different metric shape than the standard, or a distractor object. Experiment 2 served as a perceptual control for Experiment 1. Finally, in Experiment 3, we pitted topology against another surface feature that is not typically diagnostic of object category in children’s extension of novel count nouns—color.

We considered three potential hypotheses for the role of topology in children’s extension of novel labels to objects. One possibility is that, just as topology supersedes surface features like shape and color in object representation and object-based attention, topology supersedes surface features in children’s label extension. Under this possibility, we would observe a strong “topology bias” when children are asked to extend novel count nouns to novel objects. Another possibility is that topology does not play a significant role in children’s label extension. Despite the primacy of topology in vision and its potential inductive role for objects’ categories or affordances, objects’ topologies may not always be a reliable cue to objects’ categories or functions (as in the example of the cup and the mug), and changes to objects’ topologies do not necessarily change an object’s category, even if they change the object’s structure (for example, a T-shirt with a hole in it can still be worn). Under this possibility, shape should supersede topology, and we should observe the classic “shape bias” when they are pitted against each other. A third possibility is that, because topology and surface features play different roles in object representation and can provide somewhat different information about objects’ categories, we may observe *competition* between topology and shape (or color) in children’s label extensions. That is, we may observe that children view both topology and surface features as relevant dimensions along which to extend a novel label to other objects, and when faced with a forced choice between topology and surface features, may choose topology on some trials and surface features on other trials. Under this possibility, we should not observe a strong bias toward either topology or surface features in children’s extension of novel nouns.

## EXPERIMENT 1: TOPOLOGY VERSUS METRIC SHAPE

### Methods

#### Participants.

Participants were 66 2–7-year-old children (mean age = 5.0 years, range = 2.39–7.55). Parents reported their children to be female (34), male (25), or declined to report (7). Four additional children participated but were not included in analyses due to experimenter error (1), inability to speak English (1), or choosing to terminate the experiment early (2). Data were collected in the Museum of Science, Boston. All participants’ parents gave written consent for their children to participate in the study. Demographic information was not collected from participants. The Institutional Review Boards of the Boston University Charles River Campus and the Museum of Science, Boston approved all study procedures.

Our sample size was 4-5 times larger than samples obtained in previous studies using a similar method (e.g., Diesendruck & Bloom, 2003, *n* = 16; Landau et al., 1988, *n* = 12). This was because we were not certain how topology would impact children’s responses. We reasoned that a larger sample size would allow us to detect subtle effects of topology (e.g., competition between shape and topological class, which would yield similar response rates for these objects).

We also reasoned that the larger sample size would also allow us to potentially detect any effects of age on children’s responses. Previous work had revealed somewhat contradictory evidence about the extent to which shape bias develops in early childhood, with some work showing increases in shape bias between the ages of 2 and 3 years (e.g., Landau et al., 1988), and other work showing similar shape bias effects across 2- and 3-year-olds (e.g., Diesendruck & Bloom, 2003). Our age range encompassed these younger years, but also older children, because we wanted to examine the potential influence of topology in children in whom the shape bias is relatively well established, and because we were not certain whether and when such an influence would emerge in development.

#### Materials.

On each of the four trials, children viewed a set of four cardboard cutouts (each approximately 5 cm × 6 cm). Each set included a standard object, and three targets: an object of the same shape as the standard but with different topology (hole or no hole), an object with the same topology as the standard but with a different (metric) shape, and a distractor object of a different shape from the standard with “bite” taken out of its contour. The “bite” served to roughly equate the contour complexity of the object with the hole. We created shapes that were not likely to be nameable by children. The holes in the open objects (approximately 1.5 cm × 2 cm) were all differently shaped to prevent children from responding based on shared shape of the holes. We chose to construct flat cardboard cut-outs to remove the depth of the hole as an added dimension of the stimuli, which diverges somewhat from previous studies that used real-world artifacts that were unfamiliar to children. Although the stimuli were flat, children could see that the objects were three-dimensional since the objects were manipulated by human hands, moved through 3D space, and were placed on a surface such that the surface could be viewed surrounding the stimuli and through the holes in the stimuli.

**Figure 1.**
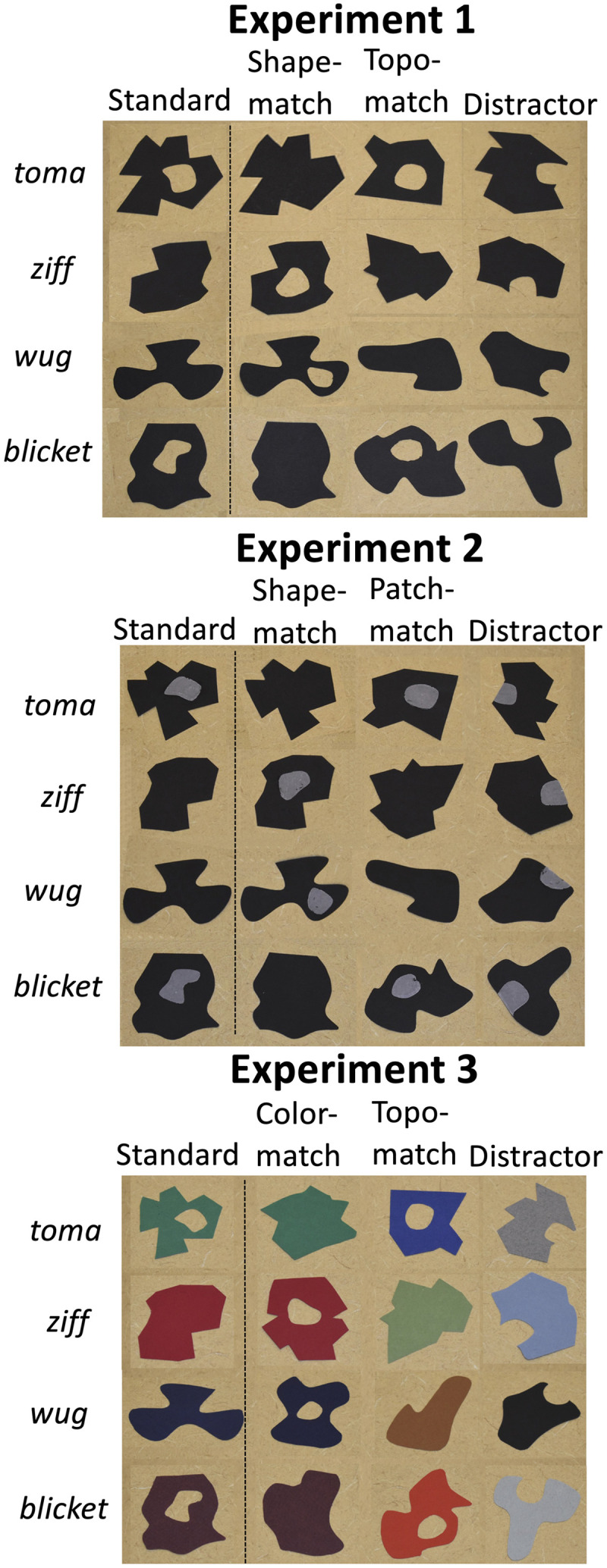
**Examples of stimuli from Experiment 1 (top panel), Experiment 2 (middle panel), and Experiment 3 (bottom panel).** Each row represents a trial from one of the possible orders. Children saw the objects placed with random orientations.

In each of the four trials, children viewed a standard object and three target objects. In two of those four trials, the standard object had a hole, and in the other two trials the standard object did not have a hole. Each child saw one of two possible sets of four trials. Across the two sets, we varied which of the objects contained the hole. Figure 1, left panel, shows one of these two sets of stimuli. Templates that can be used to construct both sets of stimuli can be accessed at https://osf.io/k3hzn/?view_only=e046ab287e6343ac9549269387748e90.

#### Procedure.

Children were tested individually in a dedicated research area in the museum. Children sat across a table from the experimenter. The experimenter told children that they were going to play a “name game”. On the first trial, the experimenter showed the standard object from the first set, and gave the object a novel name, e.g., “Look at this. It’s a *toma*. See, it’s a *toma*. This is a *toma*”. He next placed the three target objects on the table below the standard (the order of the objects was randomized across children). The experimenter then prompted children to select one of the three potential target objects, saying, “Ok, see these? Which one is also a *toma*?” After children chose, the experimenter noted their choice in a notebook, cleared the table, and then proceeded with the next three trials in the same way.

Children always chose between a shape-matched object (different in topology from the target), a topology-matched object (different in shape from the target), and a distractor object with a conspicuous divot in its outer contour. Including a distractor object on each trial allowed us to distinguish guessing from competition between the shape- and topology-matched objects. If children select the test objects at random, they should choose the distractor object in about a third of the trials. If children show competition between topology and shape, they should choose roughly equally between the shape- and topology-matched objects, and choose the distractor object only rarely.

The standard object on each of the four trials was given a unique label (*toma*, *ziff*, *wug*, or *blicket*). Whether a given label was associated with a standard object with a hole or without a hole was counterbalanced across children. All the objects were placed with random rotations (that is, there was no canonical “upright” position for any of the objects). If children attempted to touch the objects, we discouraged them from doing so (very few children attempted to touch the objects). The order of the trials was counterbalanced across participants.

### Results

Data for Experiments 1–3 are available at https://osf.io/k3hzn/?view_only=e046ab287e6343ac9549269387748e90.

We first asked whether children’s responses were different than what would be expected if children were simply choosing randomly among the three objects in each trial. Children chose the shape matched object on 126/264 trials (46.3%), the topology match on 102/264 trials (37.5%), and the distractor object on 36/264 trials (13.3%). The distribution of children’s responses was different than would be expected if children were choosing randomly (chance = 33.33%, Chi^2^(2) = 49.41, *p* < .0001). The majority of children’s choices consisted of the shape- or topology-matched object, with children choosing the distractor only infrequently.

**Figure 2.**
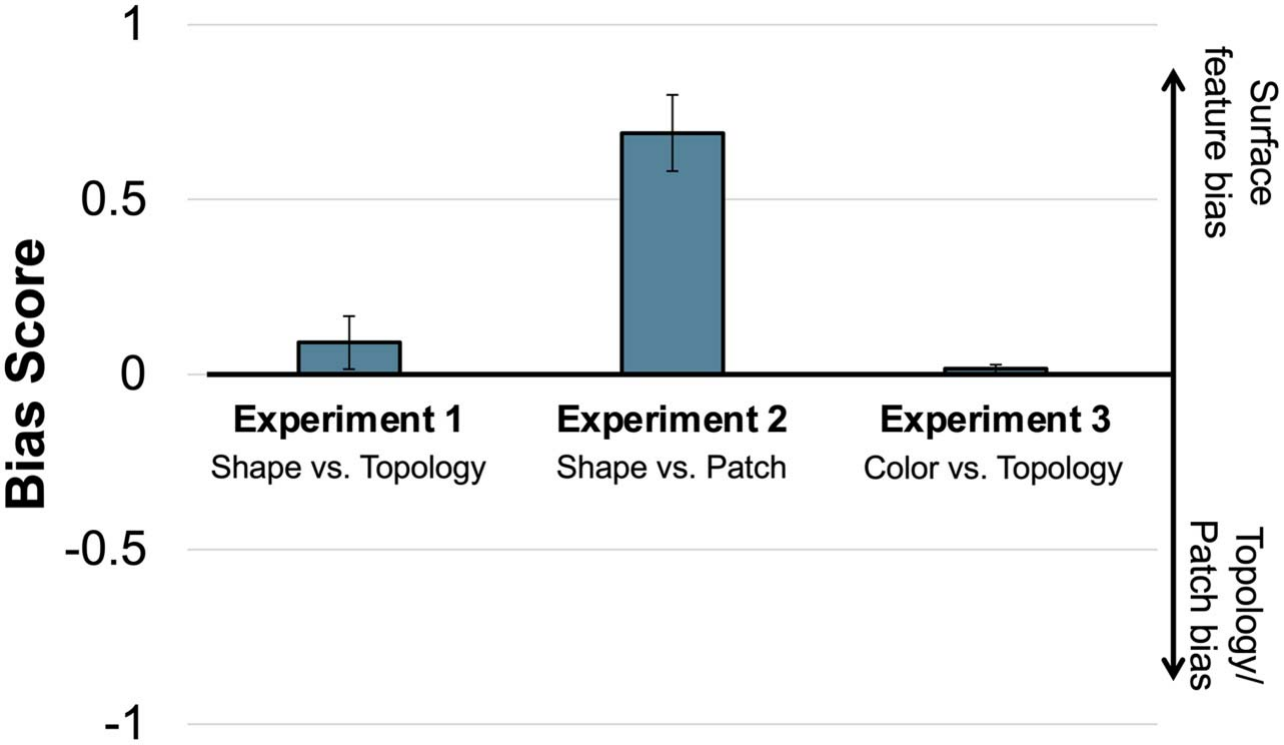
**Mean bias scores for Experiments 1, 2, and 3.** Error bars indicate ±1 standard error of the mean.

**Figure 3.**
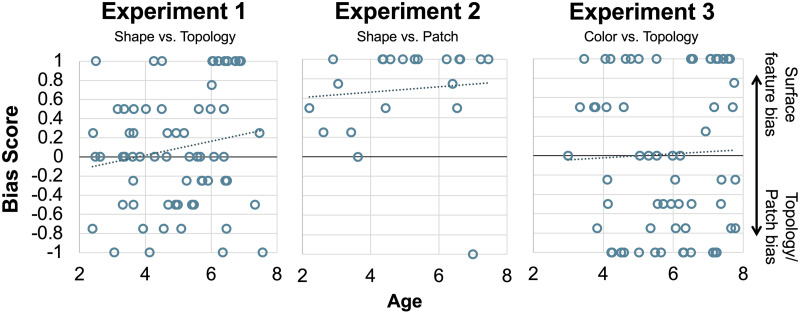
Individual children’s mean Bias Scores as a function of age for Experiments 1, 2, and 3.

To examine whether shape and topology competed with each other in children’s responses, we computed a “Bias Score” for each child, taken as the proportion of trials on which they chose the shape match minus the proportion of trials on which they chose the topology match. For example, if a child chose the shape-matched object on one trial and the topology-matched object on three trials, they would receive a Bias Score of (.25 − .75 = −.5). If a child chose the shape-matched object on two trials and the topology-matched object on two trials, they would receive a Bias Score of (.5 − .5 = 0). A positive score thus indicates a tendency to choose shape, a negative score indicates a tendency to choose topology, and a score close to 0 indicates no bias (maximum score = 1, minimum score = −1). Computing Bias Scores for each child allowed us to test the directional hypothesis that children prefer either shape (scores significantly above 0) or topology (scores significantly below 0), or show no strong preference (scores not significantly different from 0). To do so, we compared these Bias Scores to 0 using a one-sample *t* test and Bayes factor analysis. We found that children’s Bias Scores were not significantly different from 0 (*M* = .09; *t*(65) = 1.20, *p* = .235, 95% CI [−.06 .24]), with Bayes factor analysis offering support for the null hypothesis (BF_01_ = 5.13). Children’s Bias Scores were not correlated with their age in years (continuous) (*r*(65) = .167, *p* = .180). Figure 2 shows children’s mean Bias Scores. Figure 3 shows individual children’s Bias Scores as a function of age in years (continuous).

Inspection of Figure 3 provides some insights into how individual children respond across trials. Recall that a score of 1 means that a child consistently selected shape across the four trials, while a score of −1 means that a child consistently selected topology across the four trials. We found that around a quarter of the children (17/66, 26%) were “consistent choosers”. The remaining 74% of children chose topology on some trials and shape on some trials. These results suggest that, within individual children, topology competed with shape in children’s extension of novel nouns.

We next asked whether children’s responses differed when the standard had a hole versus when the standard had no hole. We separately computed Bias Scores across the two trials in which the standard had a hole and the two trials in which the standard did not have a hole and compared these using a paired samples *t* test and Bayes factor analysis. Children’s patterns of responses did not differ significantly between the two trial types (*t*(65) = −1.82, *p* = .073, 95% CI [−.38 .02]), although Bayes factor analysis offered only anecdotal support for the null hypothesis (BF_01_ = 2.10). When the standard had a hole, children’s Bias Scores were not significantly different from 0 (*M* = 0; *t*(65) = 0, *p* = 1, 95% CI [−.19 .19]) with Bayes factor offering strong support for the null (BF_01_ = 10.33). When the standard did not have a hole, children’s Bias Scores were significantly different from 0 (*M* = .18, *t*(65) = 2.11, *p* = .039, 95% CI [.01 .35]), although Bayes factor offered only anecdotal support for the alternative (BF_01_ = .81).

### Discussion

Previous work pitting shape against other surface features like color and texture found that children had a strong bias to extend novel nouns to objects that share shape over objects that share other surface features. In Experiment 1, when we pitted shape against a structural object property—topological class—we observed competition between shape and topology when children are tasked with extending novel labels to novel objects. These results suggest that topology and shape both may contribute to children’s generalization of object labels.

However, before accepting this conclusion, it is important to rule out a potential alternative explanation for children’s selections that do not exclusively rely on topology. Despite the fact that the objects visibly moved through 3D space and the texture of the table was clearly visible around the objects as well as through the objects’ holes, children may not have viewed the holes as holes, but instead as “patches” on the objects’ surfaces—essentially a figure on top of a background shape. If so, children may have matched objects based on whether or not they had this surface feature when deciding which of the objects was in the extension of the novel label. We tested this hypothesis in Experiment 2.

## EXPERIMENT 2: PERCEPTUAL CONTROL

To examine the alternative possibility that the holes in the objects in Experiment 1 were perceived as a material surface in front of a surrounding object, in Experiment 2 we replaced the holes in the stimuli from Experiment 1 with grey patches. If children’s responses in Experiment 1 were driven by perceiving the holes as figures on top of a background shape, we expected to observe similar results as in Experiment 1. However, if children in Experiment 1 were attending to topology (hole vs. no hole) as a structural property of an object, we should observe the classic shape bias effect in Experiment 2 in which the topological differences of Experiment 1 were turned into featural differences (patch vs. no patch).

### Methods

#### Participants.

Participants were 21 2–7-year-old children (mean age = 5.01 years, range = 2.21–7.46). Parents reported their children to be female (15), male (5), or declined to report (1). One additional child participated but was not included in the analyses because their parent did not provide their exact date of birth and we were therefore unable to determine their exact age. All participants’ parents gave written consent for their children to participate in the study. Children were tested in the Museum of Science, Boston, as in Experiment 1.

We anticipated collecting data from 24 children, but before we reached our planned sample size, we were forced to terminate data collection at the museum due to the ongoing COVID-19 pandemic. Our planned sample size was informed by previous work with young children demonstrating a shape bias using a similar procedure. For example, Diesendruck and Bloom (2003) included *n* = 16 children in a similar procedure and observed very large effects comparing children’s selection of the shape-matched object to chance using a one-sample *t* test (*d* > 1.5). A power analysis using the more conservative estimate of the size of the effect *d* = 1 suggested a total sample size of *n* = 10 (alpha = .05, 1-beta = .9). Diesendruck and Bloom (2003) also compared children’s responses in a condition in which the standard was labeled with a novel noun (as in the present study) with children’s responses in another condition in which their choices were expected to be evenly distributed across the three objects. They again observed a large effect size for this between-subjects comparison. A power analysis for an independent samples *t* test with effect size *d* = 1, alpha = .05, and 1-beta = .8 suggested a sample size of *n* = 17 per group. Given the novelty of our stimuli, we had opted for a target sample size of *n* = 24. However, our final sample of *n* = 21 is sufficiently powered to detect effects of interest.

#### Materials and Procedure.

Materials were identical to Experiment 1, except that the holes (and bites of distractor objects) were replaced (or filled) by gray patches in the same position and of the same shape as the holes/bites in Experiment 1 (see Figure 1, middle panel). Templates of the stimuli are available at https://osf.io/k3hzn/?view_only=e046ab287e6343ac9549269387748e90.

The procedure was otherwise identical to Experiment 1.

### Results

The distribution of children’s responses was different than would be expected by chance (chance = 33.33%, Chi^2^(2) = 86.08, *p* < .0001) and reflected the shape bias observed in previous studies (e.g., Diesendruck & Bloom, 2003; Landau et al., 1988, 1992). Children chose the shape-matched object on the majority of trials (68/84 trials, 70.8%). Of the remaining trials, children chose the patch-matched object on 10/84 trials (10.4%) and the distractor object on 6/84 trials (6.3%).

As in Experiment 1, we computed Bias Scores for each child by subtracting the mean number of trials on which they chose the patch-matched object from the mean number of trials on which they chose the shape-matched object. We found that children’s Bias Scores were significantly greater than 0 (*M* = .69; *t*(20) = 6.34, *p* < .001, 95% CI [.46 .92]), with Bayes factor offering decisive support in favor of the alternative hypothesis that children’s Bias Scores are reliably different from 0 (BF_10_ = 6493.5) (see Figure 2). Children’s Bias Scores were not correlated with their age in years (continuous) (*r*(20) = .09, *p* = .699; see Figure 3). A little over half of the children were consistent choosers (13/23, 57%), with 12 of those 13 children choosing shape on all four trials, and the majority of the remaining children biased toward shape (see Figure 3 for distribution of children’s bias scores). There was no significant difference in children’s Bias Scores on trials in which the standard had a patch versus when the standard had no patch (*t*(20) = −1.00, *p* = .329, 95% CI [−.44 .16]), with Bayes factor offering modest support for the null hypothesis (BF_01_ = 3.74). Bias Scores were significantly different from 0 both when the standard had a patch (*M* = .62; *t*(20) = 4.24, *p* < .001, 95% CI [.31 .92], BF_10_ = 80.02) and when the standard did not have a patch (*M* = .76; *t*(20) = 6.78, *p* < .001, 95% CI [.53 .99], BF_10_ = 15,625).

#### Experiments 1 and 2 Compared.

Children’s Bias Scores in Experiment 2 were significantly higher than in Experiment 1 (*t*(85) = 4.05, *p* < .001, 95% CI [.31 .89]), with Bayes factor analysis offering decisive support for the alternative hypothesis (BF_10_ = 199.12). Children’s Bias Scores were not correlated with their age in days, controlling for Experiment (*r*(89) = .09, *p* = .414).

### Discussion

In Experiment 2, when the interior region enclosed by the object changed from an empty hole into a material surface attached on it, children showed the classic shape bias—children extended novel labels to objects that shared the same shape as the standard object. These results suggest that results of Experiment 1 were not due to children perceiving the objects’ holes as figures on top of a background object, but instead suggest competition between objects’ structural properties (hole or no hole) and surface featural properties (shape) in children’s extension of novel nouns.

Most previous research on topological perception reviewed above has (implicitly) treated a hole as a 2D image region—a uniformly connected region surrounded by another region of a 2D image (e.g., 2D concentric discs forming a doughnut), and thus did not consider whether the interior region enclosed by the surrounding object was indeed seen as a background surface (a hole). A comparison between Experiments 1 and 2, however, suggests that the perception of topological properties is not based on image-level representations, but mediated by surface-level representations (i.e., surface depth order), much as motion perception, texture segregation, and object recognition are based on surface-level representations (Nakayama et al., 1995).

Experiment 2 also addressed an additional potential alternative explanation for the results of Experiment 1. In Experiment 1, because we placed the objects with random rotations, children could have been less likely to match the objects based on shape, since doing so required a bit of mental rotation (in order to match the rotated target shape to the standard). However, in Experiment 2 in which objects also were placed with random rotations, children generalized a novel name to orientation changes of the same Euclidean shape (i.e., a standard shape bias), suggesting that the rotations of the objects did not drive the diminished shape bias we observed in Experiment 1. Further, in line with the notion of Euclidean shape, this suggests that orientation is not a relevant property characterizing objects in the name generalization task.

## EXPERIMENT 3: TOPOLOGY VERSUS COLOR

In Experiment 3, we asked whether topology competes with other surface features more generally, or whether the effect is limited to metric shape, by pitting objects’ topologies against objects’ colors. Children viewed a standard object, and then were shown three target objects: one with the same color as the standard but different topology, one with the same topology as the standard but different color, and a distractor object. We considered two possible outcomes for this experiment. On the one hand, both topology and shape can provide cues to the categories or functions of artifacts, and therefore the competition observed in Experiment 1 may be specific to the case in which these two object properties are pitted against each other. On the other hand, children may focus *either* on structural properties or surface features when extending labels to novel objects, in which case we may observe competition between color and topology, similar to the competition between shape and topology observed in Experiment 1.

### Methods

#### Participants.

Participants were 64 3–7-year-olds (mean age = 5.83 years; range = 2.99–7.77). Parents reported their children to be female (27), male (26), or declined to report their child’s sex (11). Sample size for this experiment was chosen using the same criteria as in Experiment 1. Three additional children participated in the study but were excluded from the analysis due to unwillingness to finish the experiment (1), developmental disability (1), or inability to speak English and therefore to understand the task instructions (1). Children were tested at the Museum of Science, Boston, as in Experiments 1 and 2.

#### Materials and Procedure.

The materials and procedure were similar to Experiments 1 and 2, with the following exception. Each trial included one standard object and three target objects: an object with same color but different topology than the standard, an object with same topology but different color than the standard, and a distractor object with bite taken out of its contour, different in color from all the other objects in that trial (see Figure 1). As in Experiment 1, the shape of each hole varied across all of the objects to prevent children from responding based on hole shape.

As in Experiment 1, we created two sets of trials, varying which objects had the hole across the two sets. In each of the sets, there were two trials in which the standard object had a hole and two trials in which the standard object did not have a hole (see Figure 1 for an example of one of these sets). The order of the trials was counterbalanced between the participants. Stimuli were constructed from different colored carboard using the templates for Experiment 1. The colors used are shown in Figure 1.

### Results

Children’s responses were different than would be expected if they were choosing randomly between the three target objects (chance = 33.33%, Chi^2^(2) = 65.299, *p* < .0001): children selected the topology-matched object in 113/254 trials (44.48%), the color-matched object in 117/254 trials (46.06%), and the distractor object in 24/254 trials (9.44%).

As in Experiments 1 and 2, we computed a Bias Score for each child by subtracting the proportion of trials in which children selected the topology match from the proportion of trials in which children selected the color match. A positive bias score here indicates a tendency towards color, a negative bias score indicates a tendency towards topology, and a bias score closer to 0 indicates competition. Bias Scores were not significantly different from 0 (*M* = .02, *t*(63) = .16, *p* = .874, 95% CI [−.18 .21]) and Bayes factor analysis resulted in strong support for the null hypothesis over alternative hypothesis (BF_01_ = 1.499 × 10^−12^), see Figure 2. Children’s Bias Scores were not correlated with age in years (continuous) (*r*(64) = .037, *p* = .773; see Figure 3). Around half of the children consistently chose either color or topology across the four trials (31/64, 48%), with the remaining children choosing color on some trials and topology on other trials suggesting competition between color and topology (see Figure 3 for bias score distributions).

There was a small but significant difference in children’s responses on trials in which the standard had a hole compared to trials in which the standard had no hole (*t*(63), *p* = .047, 95% CI [−.501, −.003]) although Bayes factor analysis yielded only anecdotal support for the alternative hypothesis (BF_10_ = 1.441). Bias Scores across trials in which the standard object had a hole were not significantly different from 0 (*M* = −.07, t(63) = −.629, *p* = .532, 95% CI [−.293, .153]) and Bayes factor analysis showed moderate support for null hypothesis (BF_01_ = 8.381). For trials in which the standard object did not have a hole, Bias Scores also were not significantly different from 0 (*M* = .101, *t*(63) = .994, *p* = .324, 95% CI [−.102, .305]) and Bayes factor analysis again showed moderate support for null hypothesis (BF_01_ = 6.276).

#### Experiments 1 and 3 Compared.

We compared the distribution of children’s Bias Scores in Experiments 1 and 3 using a Kolmogorov-Smirnov Test. We found children’s response distributions did not vary between Experiments 1 and 3 (*p* = .372) suggesting that shape and color compete with topology in a similar manner.

Children in Experiment 3 were slightly older on average than in Experiment 1. To examine whether children’s ages impacted their responses across the two experiments, we ran an ANOVA on children’s Bias Scores with Experiment (1 and 3) as a between-participants factor and Age (in years, continuous) as a covariate. This revealed no significant effects of Experiment (*F*(1, 127) = .80, *p* = .374) or Age (*F*(1, 127) = 1.16, *p* = .284).

## GENERAL DISCUSSION

Past work has shown that objects’ surface features—particularly metric shape—play an important role in children’s extension of labels to novel artifacts (Dewar & Xu, 2007, 2009; Diesendruck & Bloom, 2003, Graham & Diesendruck, 2010; Landau et al., 1988, 1992). In a series of three experiments, we asked whether objects’ *structural properties*—specifically topology—also play a role in children’s extension of labels to novel objects. Topological properties of objects are processed earlier in perception than surface features like shape (Chen, 1985, 2005), have inductive potential for object categories, and can be associated with count nouns in the real world (Giralt & Bloom, 2000). Our goal was to examine whether and to what extent topological properties drive 3-8-year-old children’s extension of count nouns to novel objects.

In Experiment 1, when topological class was pitted against shape in a name generalization task, 3–8-year-old children’s extension of novel labels to novel objects suggested competition between shape and topology: children chose the shape- and topology-matched target objects at similar rates when asked which target object had the same label as a standard object. These results were not due to children choosing randomly—children very rarely chose the distractor object, instead selecting between the shape- or topology-matched objects. The results also were not driven by children using the shape of the hole to generalize the label, since the shape of the holes in the test and standard objects varied across all objects. We did not find any statistical differences in children’s responses when the standard object had a hole compared to when the standard object did not have a hole, suggesting that children in our sample were extending labels based on objects’ topology (with or without a hole) and not simply treating the hole as a salient feature. In Experiment 2, we further confirmed that children’s responses in Experiment 1 were driven by objects’ topologies by replacing the objects’ holes with gray patches, reversing the figural status of an interior region enclosed by the surrounding object, from an empty hole (background) to a material surface (foreground). We observed the classic shape bias effect in Experiment 2, and children’s responses in Experiment 2 were significantly different from those in Experiment 1, suggesting that in Experiment 1 topology indeed competed with shape when extending labels to novel objects. This between-experiments contrast also suggests that unlike a patch on an object, which is not an intrinsic property of the object (and thus potentially detachable), a hole is an intrinsic and ontologically parasitic property of its host object (Casati & Varzi, 1994; Kim, 2020), which is stable over changes in viewpoints, and thus counted for name extension. Finally, in Experiment 3, we pit another surface feature, color, against topology in the name generalization task, and found competition between color and topology in children’s extension of novel nouns to novel objects. Together with the results of Experiments 1 and 2, these results suggest that objects’ structural properties complete with objects’ surface featural properties in children’s extension of labels to novel objects.

Previous work has shown the primacy of topology for object perception and attention, leading to the suggestion that topological invariance is a core of object representation (e.g., Chen, 2005; Zhou et al., 2010). Nevertheless, we did not observe that topology also superseded surface features in children’s label extensions. One possibility is that the competition we observed between objects’ topologies and surface features in our name generalization task is driven by subtle differences in the inductive potential of these properties. Topological class and surface featural properties both have inductive potential for making inferences about objects’ functions, but each may provide different types of information about object category. Consider two objects with similar topologies: a coffee mug and a handbag. These two objects vary substantially in their surface features and in their basic functions (one is for holding a beverage which is then consumed, one is for carrying your personal items), but their similar topologies dictate how the objects can be interacted with by humans (e.g., both are grasped by the handle). Now consider two objects with different topologies: a needle and a pin. These two objects are very similar in shape, but their different topologies dictate how the objects can be interacted with by humans (a needle can be threaded and used for sewing, a pin cannot be threaded and is used for fastening). Both topological and shape properties of objects map onto objects’ affordances, but do not necessarily map consistently onto objects’ functions. That is, while the different functions of a pin and needle solely depend on the difference in the *topology* (and not shape) of these objects, the different functions of a mug and a handbag depend on *surface featural and material properties*, such as size and texture, and not topology. In everyday situations, we likely use *both* topology and surface features to make these different types of inferences about object categories. In our experiments, we set up a situation where children needed to use one or the other, but could not use both, and we therefore speculate that this therefore drove the competition we observed in our experiments—at least in the context of a name generalization task.

We admit that this line of argument is speculative and requires further investigation. However, it not only provides a plausible explanation of our results, but also does not conflict with extant accounts of the shape bias in the literature. The attention learning account proposes that children extract statistical associations between the linguistic input (e.g., ‘This’), labels (e.g., ‘toma’), and perceptual categories (e.g., solid, nonsolid) that exist in the real world and use these associations to guide their attention to historically relevant perceptual features for the object or artifact in question to inform about the object category (Colunga & Smith, 2008; Landau et al., 1998). According to this account, children associate metric shape with artifacts because objects that belong to same category often share shape in the real world, and therefore shape can act as a default bootstrapping mechanism for children to generalize count nouns in the absence of a prior category knowledge (Colunga & Smith, 2008; Landau et al., 1998). Like shape, count nouns also may be associated with structural features like topology in the real world and may therefore drive attention to topological properties. For example, the label “straw” is associated with elongated objects with holes through their center that can vary in color, size, and texture. Children may therefore use these past associations between labels and topology to generalize in a novel situation in which topology is a potentially relevant feature.

Another account (e.g., Diesendruck & Bloom, 2003; Markson et al., 2008) proposes that shape acts as a cue to kind membership; children generalize count nouns to objects that share the same shape because they understand that count nouns are used to refer to object kinds, and shape provides reliable information about objects’ kinds. Similarly, children also may have an expectation that topology can provide reliable information about object kinds, even when overall shape does not. For example, a straw can be used to drink from, while a chopstick cannot; a needle can be threaded, while a pin cannot; a colander can be used to drain pasta, while a bowl cannot; a tank-top can be worn comfortably, while a pillowcase cannot. Children may recognize the inductive potential of topology as they do shape, and may also expect count nouns to refer to topological properties of objects, at least under some circumstances.

Indeed, there also are plenty of cases in which objects’ topologies do not impact their function. It is possible that unless topological differences result in obvious functional differences, holes may not necessarily play a critical role in categorization. For example, both ring doughnuts and filled doughnuts are called doughnuts in spite of their topological differences. And accidentally generated holes (which are not originally the feature of the objects) usually do not influence the identity or function of objects. For example, worms can create little holes in leaves and friction can cause pinholes in T-shirts, but the introduction of these holes does not change the objects’ categorical identities. In our task, we did not provide children with explanations for the causal origin of the holes (or lack thereof) in the objects, which may have reduced the inferential potential of topology in our task. We also used flat objects whose “artifactness” might not have been as readily apparent to children (that is, children may not have readily inferred that the objects could be used for some purpose). This was by design, since we wanted to carefully control the perceptual dimensions of the stimuli. However, the structure of the stimuli may also have reduced the inductive potential of topology in the task. Future work would investigate whether providing information on specific functions of holes and/or the causal origins of holes would impact the inferential role of object topologies versus surface features in children’s object categorization.

Previous work using the name generalization task to understand children’s extension of labels to novel objects focused on very young children, with some studies finding that shape bias increases between 2 and 3 years of age (e.g., Landau et al., 1988) and other studies showing no change in shape bias in this age range (Diesendruck & Bloom, 2003). Our experiments, by contrast, included participants from a wider age range (3–8-year-olds) in order to investigate whether children’s use of objects’ structural properties versus surface featural properties for noun generalization changed over development. We investigated a wide age range because our goal was not to find the emergence of a bias, but instead to test whether topology competes with shape in children for whom the shape bias is relatively well established, and whether the extent of this competition may change over the course of development. We did not find significant developmental change in children’s bias towards topology or surface features across our sample. These results suggest that, even with extensive experience with language and object categorization, topology may play a fairly consistent role in children’s generalization of novel nouns across the lifespan. It is possible that a finer-grained examination into the younger end of our age range, or in children older than 8, could reveal subtle developmental affects that were not revealed in our study. Future work could examine this possibility.

Our results provide some potential insights into the role of topology in object representation. Previous work showed that topological class is processed earlier in vision (e.g., Chen, 1985), earlier in development (e.g., Chen et al., 2003; Chien et al., 2012; but see also Tang et al., 2021), and plays a significant role in the way objects are represented, tracked, and attended to across the lifespan (e.g., Baillargeon et al., 2012; He et al., 2015; Hespos & Baillargeon, 2001a, 2001b, 2006; Kibbe & Leslie, 2016; Rips, 2020; Wang et al., 2005; Wei et al., 2019; Wolfe & Horowitz, 2017; Zhou et al., 2010; Zhuo et al., 2003). Our results suggest that objects’ topological properties may also play a role in the formation of object categories, but topology does not necessarily take precedence over surface features in the extension of labels. More work is needed to investigate the generalizability of this finding to contexts outside of label extension in which children are asked to make inferences about objects’ categories or kinds. It is possible that the phenomenon we observed is limited to label extension in general, or to the label extension task more specifically. For example, by asking children to choose among alternatives with narrowly-defined parameters, we artificially narrowed the hypothesis space of the possible extensions of the novel nouns (see Fodor, 1972, for broader discussion). In other, more real-world scenarios, topological properties of objects may play a different role in children’s inferences about object kinds. Or, our results could reflect a more general predisposition to attend to topology as a category- or kind-relevant feature. Future work would examine these possibilities.

In this study we only tested one aspect of topology (holes). However, there are a range of other topological properties of objects, including inside/outside relationships and connectedness between objects, which may play different roles in children’s label extension. It is possible that holes may provide stronger cues to object category membership than other topological properties, perhaps because holes may be predictive of objects’ functions. Also, our manipulation of topology (presence versus absence of a hole) may have elicited a more dichotomous representation than surface featural properties of objects like shape or color, making matching on topology easier than matching on shape or color. Future research should study the role of different topological properties in children’s extension of novel objects, including examining the inductive potential of these features for object categorization, under what circumstances children may (or may not) use these features in their extension of new nouns, to what extent topological features compete with each other during label extension, and the factors that may influence competition between topology and surface features (including the relative salience of these different object dimensions).

## ACKNOWLEDGMENTS

The authors wish to thank the staff at the Museum of Science, Boston, where the data were collected. 

## AUTHOR CONTRIBUTIONS

Praveen Kenderla: Conceptualization; Formal analysis; Investigation; Methodology; Resources; Visualization; Writing – Original draft; Writing – Review & editing. Sung-Ho Kim: Conceptualization; Methodology; Writing – Review & editing. Melissa M. Kibbe: Conceptualization; Formal analysis; Methodology; Project administration; Visualization; Writing – Review & editing.

## DATA AVAILABILITY STATEMENT

Data and stimuli templates are available at https://osf.io/k3hzn/?view_only=e046ab287e6343ac9549269387748e90.

## Note

^1^ The former three transformations, called Euclidean (or rigid) transformations, preserve both shape (i.e., angle and length ratios) and size, but the latter one, uniform dilation, preserves shape alone (but not size). According to a more conservative view, Euclidean geometry is characterized by rigid transformations alone, and thus, figures are defined by both size and shape. See Izard et al. (2022) for discussion on different characterizations of Euclidean geometry in Psychology.
